# Determining the Need for Computed Tomography Scan Following Blunt Chest Trauma through Machine Learning Approaches

**DOI:** 10.22037/aaem.v9i1.1060

**Published:** 2021-01-24

**Authors:** Mohsen Shahverdi Kondori, Hamed Malek

**Affiliations:** 1 Faculty of Computer Science and Engineering, Shahid Beheshti University, Tehran, Iran.

**Keywords:** Radiography, Tomography, X-Ray Computed, Clinical Decision Rules, Decision Trees; Machine Learning

## Abstract

**Introduction:**

The use of computed tomography (CT) scan is essential for making diagnoses for trauma patients in emergency medicine. Numerous studies have been conducted on guiding medical examinations in light of advances in machine learning, leading to more accurate and rapid diagnoses. The present study aims to propose a machine learning-based method to help emergency physicians prevent performance of unnecessary CT scans for chest trauma patients.

**Methods:**

A dataset of 1000 samples collected in nearly two years was used. Classification methods used for modeling included the support vector machine (SVM), logistic regression, Naïve Bayes, decision tree, multilayer perceptron (four hidden layers), random forest, and K nearest neighbor (KNN). The present work employs the decision tree approach (the most interpretable machine learning approach) as the final method.

**Results:**

The accuracy of 7 machine learning algorithms was investigated. The decision tree algorithm was of higher accuracy than other algorithms. The optimal tree depth of 7 was chosen using the training data. The accuracy, sensitivity and specificity of the final model was calculated to be 99.91% (95%CI: 99.10% – 100%), 100% (95%CI: 99.89% – 100%), and 99.33% (95%CI: 99.10% – 99.56%), respectively.

**Conclusion:**

Considering its high sensitivity, the proposed model seems to be sufficiently reliable for determining the need for performing a CT scan.

## Introduction

A number of studies have been published, which preferred to use chest computed tomography (CT) scan rather than chest X ray (CXR) in evaluation of traumatic thoracic injuries (1, 2). It may be impossible to completely evaluate patients and provide rapid medical services when they go to emergency departments due to limitations in time, human resources, and equipment, particularly during natural disasters with a high number of visits. In such situations, the use of clinical decision rules may be very effective in accelerating the decision-making process and can determine the priority of caring for patients and accelerate the discharge of those who do not need further care (3). Evidence-based indications for CT scan in blunt thoracic trauma have not been extensively reviewed. In an attempt in this regard, Safari et al. showed that cases with normal CXR may skip chest CT scan (4). Accordingly, the present study proposes a model to predict whether a chest CT scan is necessary, using machine learning and artificial intelligence tools and a dataset collected from patients who underwent chest CT scans. 

## Methods

***Dataset***

The dataset consisted of the data of 1000 trauma patients who referred to Shohadaye Tajrish and Imam Hossein Hospitals, which are two large trauma research centers in Tehran, from January 2017 to July 2018. All of the patients underwent initial examinations and CT scans. The data were collected from adult patients at ages above 18 years and included patients’ personal information (i.e., age and gender), incident details, trauma mechanism (either high or low energy), vital signs (i.e., heart rate, respiratory rate, blood pressure, and oxygen saturation), level of consciousness, clinical examination and history taking findings (including dyspnea, respiratory sounds, reduced cardiac sounds, chest wall deformity, distracting pain, generalized tenderness, chest wall tenderness, chest wall abrasion, crepitation, jugular venous pressure (JVP), and chest wall pain), CT scan findings, X-ray images, and sonography results. Table 1 provides the data.

***Preprocessing***

In the first preprocessing stage, a number of dataset fields were found to be irrelevant and were excluded, including gender and transportation to the hospital, as shown in column 3 of table 1. It should be noted that the exclusion of irrelevant data increases the model’s accuracy. Glasgow coma scale (GCS) field, which indicates the level of consciousness was also excluded as it divided patients into conscious and unconscious patients. The items excluded in this stage are shown in column 3 of table 1.

In the second stage, preprocessing methods used in machine learning algorithms were applied to the data. Then, irrelevant or non-effective items obtained in the second preprocessing stage based on model training results, including O2 saturation and hemoglobin level, were excluded from the dataset. Then, X-ray and sonography data were excluded, since they had a high correlation with the target value, as shown in column 4 of table 1. This is further explained below. 

Before performing the learning process with the remaining items, some categorical data, including chest CT scan findings and type of high energy trauma, were quantized using the one-hot vector method (5), as presented in column 2 of table 2. The one-hot vector transforms categorical data into binary values, allowing for building a better model through machine learning methods. 

In the third stage, the remaining data were reviewed by an expert, excluding the medically irrelevant fields and CT scan-requiring fields from the dataset, as shown in column 5 of table 1. Then, a number of items that were deemed to have the same implications by the expert were integrated, as provided in column 7 of table 1. Thus, only column 7 remained for the learning process. Then, machine learning algorithms were trained using the remaining data. 

In dealing with trauma patients, some signs necessitate CT scans, regardless of other conditions. For example, a chest wall deformity requires the medical team to perform a CT scan. The GCS level is another sign that leads physicians to prescribe CT scans – if a patient is not conscious, a CT scan must be performed. Thus, these items were also excluded from the dataset for model training. 

***Machine learning algorithms***

Machine learning is one of the most commonly employed artificial intelligence classes. It adjusts and discovers practices and algorithms by which computers and systems can learn. Classification account for a set of machine learning algorithms. The main objective of classification algorithms is to classify data into distinct groups that can detect new data. Classification methods include the support vector machine (SVM), logistic regression, Naïve Bayes (6), decision tree, multilayer perceptron (four hidden layers)(7), random forest, and K nearest neighbor (KNN) (8). Such methods have advantages and disadvantages, and the best method to address the problems should be chosen based on the specific problem and its requirements. The decision tree approach was chosen in the present study as it provides more explanation for the results, which was importance in this study.

***Decision tree***

The decision tree approach is a decision support tool that uses trees for modeling. Decision trees are typically employed in different operations, such as decision analysis, to find the best strategy to classify data. A condition is investigated in each node of a decision tree. The algorithm follows one of the two branches of a node based on whether the condition is met. This continues until a leaf is reached. Finally, decisions are made based on the number of each class of samples in a given leaf. Particularly, after investigating the entire conditions on the input data in the proposed problem, the algorithm will produce a positive outcome if the number of training samples that suggest performing a CT scan is higher than those that do not suggest performing a CT scan; otherwise, it will produce a negative outcome. Each move from a node to another adds a unit to the tree depth. The tree depth is a parameter that should be either identified during the learning process, or chosen based on the optimal depth determined using optimal depth identification methods. 

***Data Segmentation***

In a machine learning algorithm, data are segmented into training, validation, and test data. It should be noted that classification should be performed randomly. Accordingly, 60%, 20%, and 20% of the data were selected as training, validation, and test data, involving 600, 200, and 200 samples, respectively. The SVM, logistic regression, Naïve Bayes, decision tree, multilayer perceptron (four hidden layers), random forest, and KNN algorithms were applied to the data. Then, the models were evaluated using the validation data. The validation results showed a higher accuracy for the decision tree algorithm. Thus, the decision tree model was adopted. Then, 70% of the data were used to find the optimal depth, while the remaining 30% were employed as the test data. 

***Evaluation criteria***

An evaluation criterion should be used to compare machine learning algorithms and detect their efficiency. The present study employed accuracy as the evaluation criterion. Then, sensitivity was used to determine the optimal tree. In general, sensitivity is of great importance in analyzing medical data, since it represents how accurate a model is in diagnosis. 

## Results

The above-mentioned machine learning algorithms were investigated by the criterion of accuracy. Table 2 provides the training and validation accuracy of different machine learning algorithms**.** As can be seen, the decision tree algorithm had a higher accuracy compared to other algorithms. It should be noted that the results were obtained after excluding the X-ray and sonography data. In fact, the idea is to propose a model that can be employed even without X-ray and sonography equipment. 

Table 3 shows the accuracy of the proposed decision tree in different depths of the model. As can be seen, a decision tree depth of 7 was chosen. After choosing the decision tree, the tree’s depth should be measured as a parameter. The optimal tree depth was selected using the training data based on Table 4.

**Figure 1:**
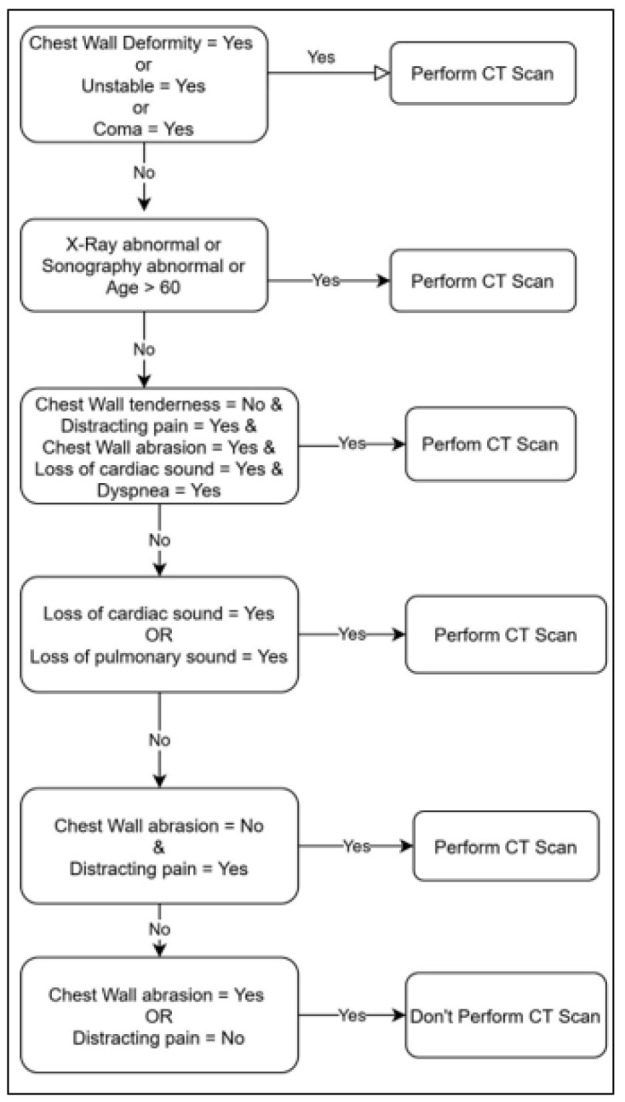
The final model obtained by re-including the excluded data.

Finally, a rule (algorithm) was obtained to be proposed to emergency physicians based on the obtained model, application of X-ray and sonography data, and incorporation of the data that were excluded from the procedure by the expert. Figure 1 presents the final model.

## Discussion

In this work an interpretable machine learning model was introduced to help emergency physicians to prevent performance of unnecessary CT scans for chest trauma patients. Due to the simplicity of the model, it is a very good choice for patient classification in order to prevent the crowding problems in critical conditions such as natural disasters like earthquakes, floods, and volcanoes. This model has good accuracy and high generalizability due to being usable in the presence or absence of sonography and x-ray results. The model, which is the final and pruned model of the decision tree, can be easily implemented in the rule diagram. 

Shapley value (9) is an analytical method in game theory, which is used in machine learning in order to increase the interpretability of models (10). The Shapley value explores the hypothesis space by considering the presence or absence of each parameter. Finally, the contribution of each parameter to the accuracy of the model is obtained as a result. In order to evaluate the sensitivity of the model to each parameter, we analyzed each of the parameters used in the model to find out the impact of each parameter on model output magnitude. In Figure 4, X-axis represents the effect of each parameter on the accuracy of the model and Y-axis represents the order of importance of the model parameters. As can be seen, GCS categories, age, and loss of pulmonary sound have the most impact on the results of the model in detecting the correct classes.

It was demonstrated that the proposed model’s sensitivity is high in identifying cases for which CT scan should be performed, and its specificity is acceptable. The model is proved to be effective with high reliability in reducing the number of patients that need CT scans. 

**Table 1:** The patient data of the dataset

**Field Name**	**Data type**	**Deleted in step one**	**Deleted in step two**	**Deleted by expert comment**	**Change@**	**Merge**	**Used** ^#^
Chest X-Ray	binary			*×*			✓
Chest X-Ray	Binary	*×*					
Age	Numerical	*×*					
Gender	Binary	*×*					
Fast Sonography	Binary			*×*			✓
Drug history	Binary	*×*					
Chest Wall Deformity	Binary			*×*			✓
Transfer to hospital	Categorical	×					
Chest Wall Tenderness	Binary						✓
Systolic blood pressure	Numerical			*×*		◆	
Distracting Pain	Binary						✓
Diastolic BP	Numerical			*×*		◆	
Loss of Cardiac Sound	Binary						✓
Glasgow coma scale (GCS)	Numerical	*×*					
Chest Wall Abrasion	Binary						✓
Respiratory rate	Numerical			*×*		◆	
Generalized Tenderness	Binary						
O2 Saturation	Numerical		*×*				
Chest Wall Pain	Binary						✓
High energy trauma	Binary		*×*				
Medical History	Binary						✓
High energy trauma	Categorical		×		×		
Heart rate	Binary		×			◆	
Dyspnea	Binary						✓
Chest CT Scan	Binary						✓
Tachypnea	Binary						✓
Hemoglobin level	Numerical		*×*				
Pulmonary Sound*	Binary						✓
Chest CT Scan finding	Categorical		*×*				
Crepitation	Binary						✓
Trauma mechanism	Categorical			*×*			
JVP enlargement	Binary						✓
Age categories	Binary						✓
Unstable hemodynamics	Binary					◆	✓
GCS categories	Binary			*×*			✓

^@^: Change to one hot; #: used for final model, *: Loss of pulmonary sound; BP: blood pressure; JVP: jugular vein pressure; GCS: Glasgow coma scale; CT: computed tomography.

**Table 2:** The accuracy of different models in training and validation phases

**Model list**	**Accuracy (95% CI)**	
	**Training**	**Validation**
Support Vector Machine	84.6 (79.87 - 89.33)	80.5 (74.62 – 86.38)
K Nearest Neighbor (k = 5)	81.33 (76.38 - 86.28)	76.5 (71.23 - 81.77)
Logistic Regression	82 (74.33 – 89.66)	77 (69.22 – 84.78)
Random Forest	85.33 (80.01 - 90.65)	80.5 (74.53 - 86.47)
Naive Bayes	80.5 (73.44 - 87.56)	74 (66.18 - 81.82)
Multilayer Perceptron (3 hidden layers)	85 (81.67 – 88.33)	82 (77.97 – 86.03)
Decision Tree (Depth = 7)	87 (84.12 - 89.88)	85 (81.58 – 88.42)

**Table 3:** Different decision tree depths and accuracies obtained on the test, validation, and sensitivity data

Depth*	**Accuracy (95% CI)**		**Test Sensitivity**
	**Training **	**Test**	
3	81.85 (78.75 – 84.95)	83.33 (79.53 – 87.13)	95.45 (92.55 – 98.35)
4	83.14 (80.08 – 86.2)	83.66 (79.8 – 87.52)	97.47 (94.66 – 100)
5	84.28 (81.27 – 87.29)	84 (80.28 – 87.72)	95.95 (93.43 – 98.47)
6	85.28 (82.41 – 88.15)	84.33 (80.66 – 88)	95.45 (93.44 – 97.46)
7	86.57 (83.79 – 89.35)	85 (81.51 – 88.49)	97.47 (95.59 – 99.35)
8	87.28 (84.56 – 90)	85.33 (81.73 – 88.93)	97.47 (95.55 – 99.39)
9	88.14 (85.52 – 90.76)	83.66 (79.94 – 87.38)	93.93 (91.96 – 95.9)
10	88.85 (86.25 – 91.45)	84.66 (80.73 – 88.59)	94.94 (92.88 – 97)

*: Model depth. Data are presented with 95% confidence interval.

**Table 4:** The decision tree results with and without considering the chest X-ray and sonography findings

**Model**	**Accuracy ** **(95% CI)**		**Test**	
	**Training**	**Test**	**Sensitivity**	**Specificity**
With	99.95 (99.28 – 100)	99.91 (99.1 – 100)	100 (99.81 – 100)	99.33 (99.1 – 99.56)
Without	85.5 (93.32 – 87.68)	84.66 (81.9 – 87.42)	98.96 (98.05 – 99.87)	77.83 (72.49 – 83.17)

Data are presented with 95% confidence interval.

**Figure 2:**
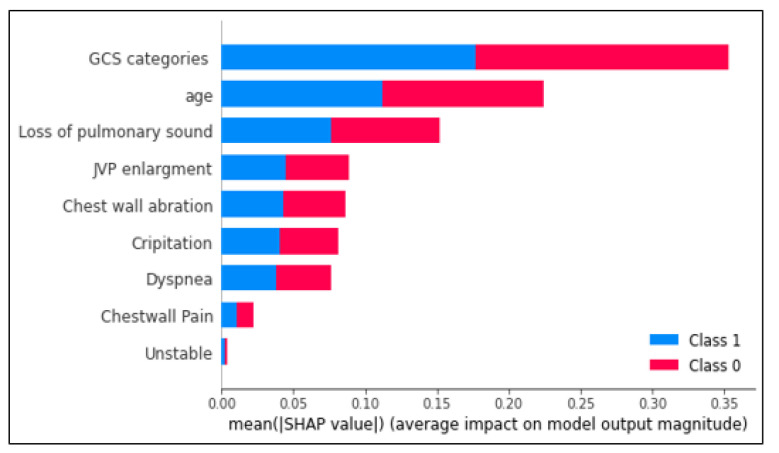
The contribution of parameters to model accuracy. JVP: jugular vein pressure; GCS: Glasgow coma scale.

## Conclusion

Trauma poses a challenge in emergency departments regarding providing early care for patients. Proper hospital equipment is required to perform CT scans on trauma patients and its cost is high. The present study proposed a decision tree-based model to determine whether a CT scan is necessary early on. Considering its high sensitivity, the proposed model seems to be sufficiently reliable in determining the need for performing a CT scan. 

## Support

Nothing to declare
